# Identification and validation of RNA‐binding protein‐related gene signature revealed potential associations with immunosuppression and drug sensitivity in glioma

**DOI:** 10.1002/cam4.4248

**Published:** 2021-09-05

**Authors:** Zhuohui Chen, Haiyue Wu, Haojun Yang, Yishu Fan, Songfeng Zhao, Mengqi Zhang

**Affiliations:** ^1^ Department of Neurology Xiangya Hospital Central South University Changsha China

**Keywords:** drug sensitivity, glioma, immune infiltration, prognostic value, RNA‐binding protein, tumor immunity

## Abstract

**Background:**

Glioma is the most common central nervous system tumor in adults, and a considerable part of them are high‐degree ones with high malignancy and poor prognosis. At present, the classification and treatment of glioma are mainly based on its histological characteristics, so studies at the molecular level are needed.

**Methods:**

RNA‐seq data from The Cancer Genome Atlas (TCGA) datasets (n = 703) and Chinese Glioma Genome Atlas (CGGA) were utilized to find out the differentially expressed RNA‐binding proteins (RBPs) between normal cerebral tissue and glioma. A prediction system for the prognosis of glioma patients based on 11 RBPs was established and validated using uni‐ and multi‐variate Cox regression analyses. STITCH and CMap databases were exploited to identify putative drugs and their targets. Single sample gene set enrichment analysis (ssGSEA) was used to calculate scores of specific immune‐related gene sets. IC50 of over 20,000 compounds in 60 cancer cell lines was collected from the CellMiner database to test the drug sensitivity prediction value of the RBP‐based signature.

**Results:**

We established a reliable prediction system for the prognosis of glioma patients based on 11 RBPs including THOC3, LSM11, SARNP, PABPC1L2B, SMN1, BRCA1, ZC3H8, DZIP1L, HEXIM2, LARP4B, and ZC3H12B. These RBPs were primarily associated with ribosome and post‐transcriptional regulation. RBP‐based risk scores were closely related to immune cells and immune function. We also confirmed the potential of the signature to predict the drug sensitivity of currently approved or evaluated drugs.

**Conclusions:**

Differentially expressed RBPs in glioma can be used as a basis for prognosis prediction, new drugs screening and drug sensitivity prediction. As RBP‐based glioma risk scores were associated with immunity, immunotherapy may become an important treatment for glioma in the future.

## Graphical abstract

We established and verified a prediction system for the prognosis of glioma patients based on 11 RBPs, and proposed several drugs with therapeutic potential on the basis of the differentially expressed genes. RBP‐based risk scores have a close relationship with immune cells, immune function, and drug sensitivity. Immunosuppression in glioma patients indicates that immunotherapy may become a promising treatment for glioma in the future.

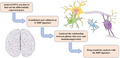

## 1 BACKGROUND

RNA‐binding proteins (RBPs) comprise a large family of more than 2000 proteins that bind to double or single‐stranded RNA through RNA‐binding domains (RBDs) to form ribonucleoprotein complexes.^1^ They participated in the regulation of cell metabolism and functions, and played a key role in cell proliferation, cell differentiation, and carcinogenesis. It has been found out that some RBPs acted as promoters for oncogenesis and tumor growth in non‐small cell lung cancer, glioblastoma (GBM), several leukemias, and hepatocellular carcinoma.^2^, ^3^, ^4^ And some RBPs played a role as tumor suppressors in breast cancer, hepatocellular carcinoma et al.^2^, ^5^, ^6^ For instance, Lin28 was found to be a highly conversed RNA‐binding protein that was involved in both important biological processes and tumor progression and metastasis of various human cancers.^7^ By antagonizing epithelial‐mesenchymal transition (EMT)‐associated alternative splicing, A‐Kinase Anchor Protein (AKAP8) suppressed breast cancer metastasis.^8^ Previous studies have shown that RBPs were significant in post‐transcriptional regulation and the activity of the RBP‐RNA network had a causal relationship with cancer development.^9^ RBP also played an indispensable role in metabolic activities of the central nervous system (CNS) and the occurrence and development of CNS diseases. For example, antibodies to heterogeneous nuclear ribonucleoprotein A1 (hnRNP A1) which was an RBP overexpressed in neurons, was found in multiple sclerosis patients, suggesting that anti‐hnRNP A1 antibodies were involved in neurodegeneration in the immune‐mediated disease of CNS.^10^ The insulin‐like growth factor‐2 mRNA‐binding protein 1 (IGF2BP1), which was essential for both embryogenesis and carcinogenesis, was associated with poor overall survival and metastasis in a variety of human cancers by acting as a post‐transcriptional fine‐tuner.^11^ Although the functions of RBPs were gradually uncovered, more studies on the relationship between RBP and the pathogenesis of glioma are needed, and it is still difficult for current studies to provide good guidance for clinical diagnosis and treatment.

Glioma is a kind of tumor of the central nervous system that originates from the glial cells, which are the supportive cells in the brain. It is the most common type of primary tumor of the CNS. Recently, The World Health Organization has made molecular parameters in addition to histology to define gliomas.^12^ Gliomas are classified into grades I–IV pathologically, and the prognosis of gliomas with different grades presented varied prognoses in the clinical. Glioblastoma is a kind of Grade IV glioma and the median survival of treated patients with glioblastoma is about 15 months, while although low‐grade gliomas (LGG) seem to have longer median survival, they tend to progress to higher grade gliomas of grade III and grade IV.^13^, ^14^ Glioblastoma (Grade IV) is the most malignant type with the short survival time of patients and the limited available treatments, mainly non‐glioma‐specific radio‐chemotherapy with the alkylating agent after maximal safe resection.^15^ Lower grade gliomas (Grade II and III) occur in the younger population and have a better prognosis. In the classification for LGG proposed by WHO in 2016, several molecules including Isocitrate dehydrogenase (IDH) mutation, IDH wildtype, and 1p/19q were included in the criteria for LGG molecular subgroup classification for the first time.^12^ Molecular diagnosis is conducive to accurate treatment and there are a lot of studies underway in this area, but there is still a long way to go.^16^, ^17^ Compared to histology, molecular parameters can be earlier detected, which facilitates earlier diagnosis and treatment, leading to the potential to improve patients’ prognosis. Researchers have found that the expression of RBPs was closely related to the malignancy of glioma. Some RBPs played the role of tumor suppressors and their expression often decreases in human glioma tissue and cell lines, while some others who acted as promoters are upregulated. For instance, overexpressed LARP4B could significantly inhibit proliferation and was associated with the increased expression level of CDKN1A and BAX.^18^ However, the knockdown of PCBP2 inhibited glioma growth both in vitro and in vivo by suppressing cell‐cycle and the apoptosis mediated by caspase‐3, showing that PCBP2 may act as a promoter of glioma development. Some RBPs regulated cellular activity in glial cells through synergistic action with other RBPs or RNA. MOV10, circ‐DICER1, which were important in regulation for cell migration, viability, and tube formation, were both found upregulated in glioma‐exposed endothelial cells (GECs), and silencing of both of them have a better effect than silencing one of them alone.^19^ Moreover, the upregulated RNA‐binding protein in GECs, FUS, participated in the feedback loop of FUS/circ_002136/miR‐138‐5p/SOX13 and regulated the angiogenesis in glioma.^20^ ZRANB2 was also overexpressed in glioma cells and tissue, being a part of the ZRANB2/SNHG20/FOXK1 axis which was essential in regulating the vasculogenic mimicry formation of glioma. KHSRP played a key role in metastasis of non‐small cell lung cancer and may be a prognostic predictor.^3^ FXR1 was another important RBP for non‐small cell lung cancer (NSCLC) development, and research has found that its expression was a candidate biomarker for poor survival in a variety of solid tumors, including NSCLC. Since the function of RBPs is closely related to tumor‐related biological activities such as protein synthesis, cell proliferation, and immune function, RBP can relatively reliably reflect the progress of tumor, which is of great significance for the prognosis of patients.^21^

To demonstrate and annotate the integrative roles of RBPs in gliomas, in our present study, we identified the aberrantly expressed RBPs of prognostic based on Low‐Grade Glioma and Glioblastoma Multiforme cohorts from the TCGA database. Gene ontology (GO) and the Kyoto Encyclopedia of Genes and Genomes (KEGG) were performed to annotate the function. We then identified the hub genes and constructed an 11‐RBPs signature to predict the clinical outcome of glioma, which showed a good prediction efficiency in both the TCGA training cohort and CGGA validation cohort. We also screened some drugs which are potential for glioma treatment and their target protein, as well as performed a correlative analysis of glioma risk score and drug sensitivity to select drugs that can be used to more precisely treat high‐risk or low‐risk gliomas. Immune score analysis showed that high‐risk groups had a higher gene enrichment in both anti‐tumor and immune‐suppressive gene sets, highlighting the potential therapeutic targets in both RBPs and tumor microenvironment immunomodulation.

## 2 MATERIALS AND METHODS

### 2.1 Datasets acquisition

In the present study, we integrated three cohorts including Low‐Grade Glioma (LGG) and Glioblastoma Multiforme (GBM) from The Cancer Genome Atlas (TCGA) datasets (*n* = 703); and a Chinese cohort from the Chinese Glioma Genome Atlas (CGGA) database (*n* = 325).^13^, ^22^ The RNA‐seq data and clinical information from TCGA were collected to perform gene differential expression analysis and construction of prognostic gene signature, after which the data from CGGA were used for validation. The accessible websites of TCGA and CGGA are https://portal.gdc.cancer.gov/ and http://www.cgga.org.cn/, respectively.

### 2.2 Gene differential expression analysis

The RNA binding proteins (RBPs) were identified from the following resources: SONAR, Gerstberger, Poly(A)‐binding protein, the Gene Ontology project, XRNAX, and CARIC. Among the 1542 RBPs, differentially expressed genes (DEGs) were identified using transcriptome data from TCGA. To perform the analysis, the Limma package for R was used to compare the expression between normal tissues (*n* = 5) and tumor tissues (*n* = 698).^23^ Genes with False Discovery Rate (FDR) lower than 0.05 and |logFC| > 0.5 were accepted for further analysis.

### 2.3 Gene correlation analysis and network construction

STRING database (http://string.embl.de/) is a widely used tool to predict the functional interactions between proteins by integrating direct interaction (physical interaction) and indirect interaction (functional association) information from accessible experiments data.^24^ In the present study, we input all the DEGs into the database to acquire the correlation coefficient between every two proteins. After that, the correlation data were imported into Cytoscape 3.8.0 for reconstruction and visualization.^25^ To determine the hub genes of DEGs, we reanalyzed the correlation data by plug‐in MCODE and determined several subnetworks. The top three scored subnetworks were visualized in our study.

### 2.4 Gene signature construction and validation

Univariate and multivariate Cox regression analysis was successively conducted to determine genes highly correlated with overall survival and survival status using TCGA datasets. The FDR filter of univariate Cox regression analysis was 0.001 and Hazard Ratio (HR) filter was 1.5 or 0.5, thus selecting out 28 genes for gene signature construction in multivariate Cox regression analysis. As a result, we constructed an 11‐gene signature that can be used to calculate risk scores for each patient. The formula can be as follows:

(1) $$\text{riskScore}=\sum_{i=1}^{n}\text{Coef}\left(X_{i}\right)\times \exp\left(X_{i}\right)$$

Coef(*X_i_*) was considered as the coefficient of each RBP *X_i_*, and Exp(*X_i_*) was considered as the expression levels of these genes. Based on the formula, the risk scores of each patient in both TCGA and CGGA datasets were calculated; and patients in each dataset were divided into low‐ or high‐ risk groups based on the median risk score cutoff.

### 2.5 Gene functional annotation analysis

The functional annotation of genes acquired from subnetwork or DEGs was performed using the ClusterProfiler package for R, including Gene Ontology (GO) and Kyoto Encyclopedia of Genes and Genomes (KEGG) pathway analysis.^26^ To figure out whether immune cell infiltration and function differed between low‐ and high‐ risk patients, we also performed single‐sample gene set enrichment analysis (ssGSEA) using GSVA package for R to calculate scores of specific immune‐related gene sets in each patient, and then integrated the score based on their risk clustering.^27^

### 2.6 Putative drugs identification

To determine the possible small molecules targeting DEGs of our study, The Connectivity Map (CMap) database (https://portals.broadinstitute.org/cmap/) was used to perform the prediction. The enrichment score represents the effects of a drug on the input gene set. The negative value was considered to hold the capacity to reverse gene expression, which in our cases, to be a candidate anti‐tumor drug. The filters for small molecule compounds were *p* value <0.05 and enrichment scores <−0.85. To further determine the potential target proteins of the predicted drugs, drugs with *p* value <0.05 were selected and analyzed using the STITCH database (http://stitch.embl.de/). The STITCH database^28^ can predict the interactions between drugs and proteins based on currently published studies and experiments, and other predicting databases together.

### 2.7 Association analysis of RBP‐based riskScore and drug sensitivity

The transcriptome for 60 cancer cell lines and IC50 of over 20,000 compounds were downloaded from the CellMiner database v2.5 (https://discover.nci.nih.gov/cellminer/home.do).^29^ To enhance the relationship of RBP‐based riskScore and clinical application, only FDA approved drugs and drugs under clinical trials were included in the analysis. Spearman correlation analysis was performed to determine the correlation between RBP‐based riskScore and drug sensitivity. Correlations with |cor| > 0.3 and *p* value <0.01 were considered as statistically significant.

### 2.8 The validation and landscape of protein expression, mutation, copy number alteration, and structural variant

To validate the differential gene expression of the 11 RBPs, we searched The Human Protein Atlas (HPA) database (http://www.proteinatlas.org/) to acquire the public immunohistochemistry (IHC) figure of these RBPs in both normal brain tissues and glioma tissues. An oncoprint presenting the mutation, copy number alteration, and structural variant landscape of the 11 RBPs in 14 glioma‐related datasets was constructed using the cBioPortal database (http://www.cbioportal.org/).

### 2.9 Statistical analysis

All the statistical analysis and figure construction were completed in R software version 3.6.0 (https://www.R‐project.org/) using packages including Ggplot2 and Ggpubr. KM plotter was performed and analyzed using a log‐rank test. Linear correlation analysis between the expression of immune checkpoint and risk score was implemented by calculating Spearman's coefficient. Nomogram enrolling statistically significant clinical variants in multivariate Cox regression analysis was constructed. C‐index was calculated to determine the concordance between prediction and actual diagnosis. Kruskal–Wallis *H*‐test was used to compare the difference between groups. In all the statistical analyses, *p* value <0.05 was considered statistically significant.

## 3 RESULTS

### 3.1 The differential expression of a variety of RBPs is closely related to the incidence of glioma

It has been reported that an amount of RNA‐binding proteins’ expression was changed in glioma, indicating that these RBPs may be correlated to glioma development.^19^, ^30^, ^31^ In previous studies, RBPs could participate in glioma development in various ways. For instance, RBPs could promote glioma by affecting vasculogenic mimicry formation, and suppressing it by preventing let‐7 target gene silencing, which contributed to cell differentiation.^32^, ^33^

**FIGURE 1:**
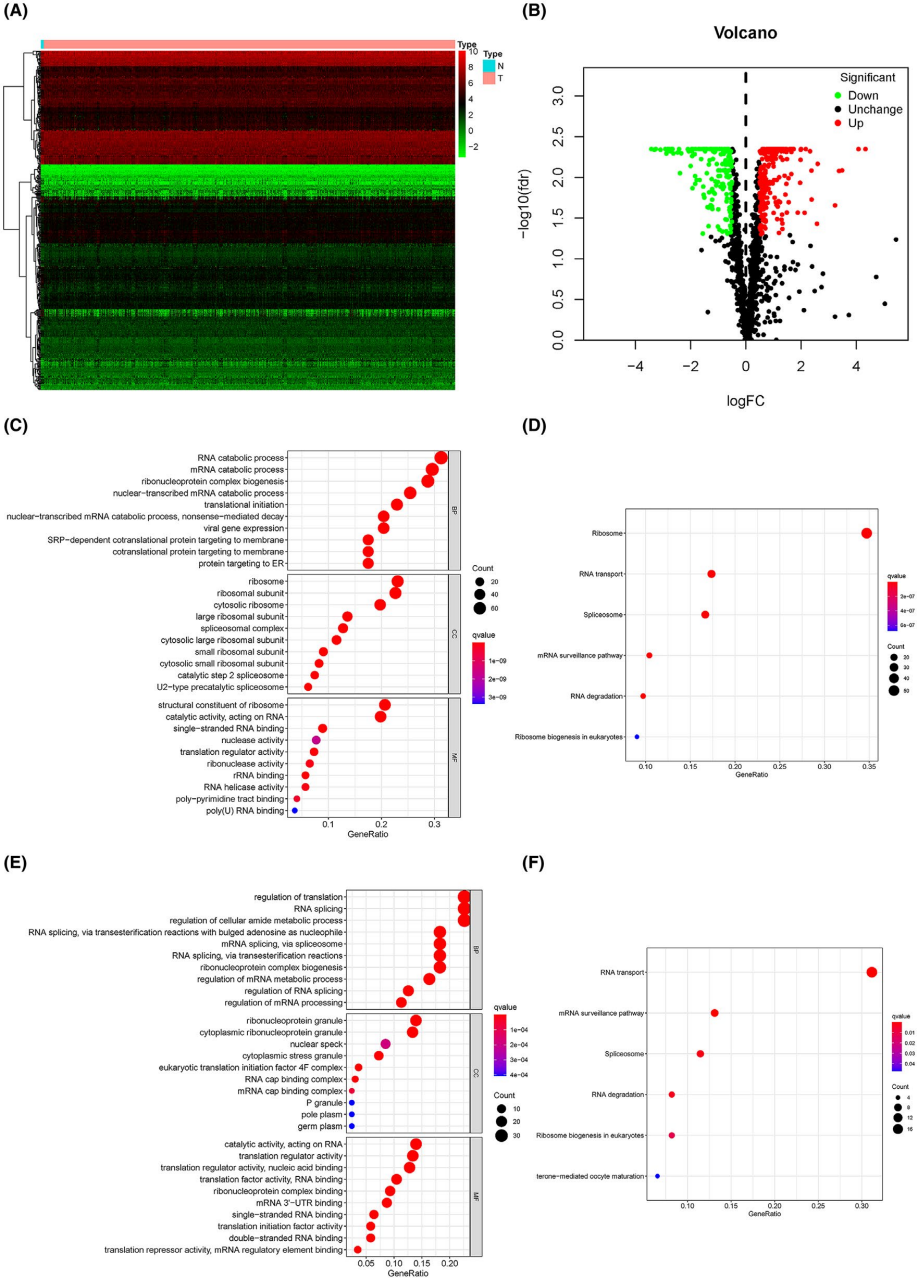
The expression of a variety of RBPs is closely related to the incidence of glioma. (A) Heatmap of RNA‐sequencing data analysis of 698 glioma patients and 5 normal samples from TCGA‐GBM and TCGA‐LGG of TCGA. (B) Volcano map of differentially expressed RBP genes to screen out ones with significantly different expressions. (FDR ≤ 0.05, |logFC| > 0.5) (C, E) Bubble maps of gene ontology obtained by GO and KEGG analysis of selected upregulated and downregulated genes. (D, F) Bubble maps and barplots of gene ontology obtained by GO and KEGG analysis of selected upregulated and downregulated genes. BP, biological process; CC, cell cycle; MF, molecular function

To find out the RBPs which expressed differentially between normal samples and the ones with glioma, we downloaded RNA‐sequencing data of 698 glioma patients and 5 normal samples from TCGA‐GBM and TCGA‐LGG (Low‐grade glioma) of The Cancer Genome Atlas (TCGA). First, we performed the analysis of RBPs’ expression on the basis of RNA‐sequencing data (Figure 1A) and then made a volcano map to select genes that had a significantly different expressions between the normal group and the glioma group (Figure 1B, FDR ≤ 0.05, |logFC| > 0.5). Two hundred and fifty‐five upregulated and 185 downregulated genes were screened out, gene ontology (GO) and Kyoto Encyclopedia of Genes and Genomes (KEGG), were performed to confirm that these genes are closely related to RBPs. The upregulated RBPs were mostly associated with the ribosome and RNA catabolic process, which was consistent with increased tumor cell division and protein synthesis. (Figure 1C,D) Moreover, most of the downregulated RBPs were connected with RNA transport and translation regulation, suggesting that they may be associated with abnormal gene expression in tumor cells, including overexpression of oncogene and inhibited expression of tumor suppressor gene. (Figure 1E,F) As a result, it can be confirmed that the genes selected by us are closely related to the differential expression of RBP between normal samples and glioma samples.

### 3.2 The RBPs closely associated with glioma is primarily associated with ribosome and post‐transcriptional regulation

**FIGURE 2:**
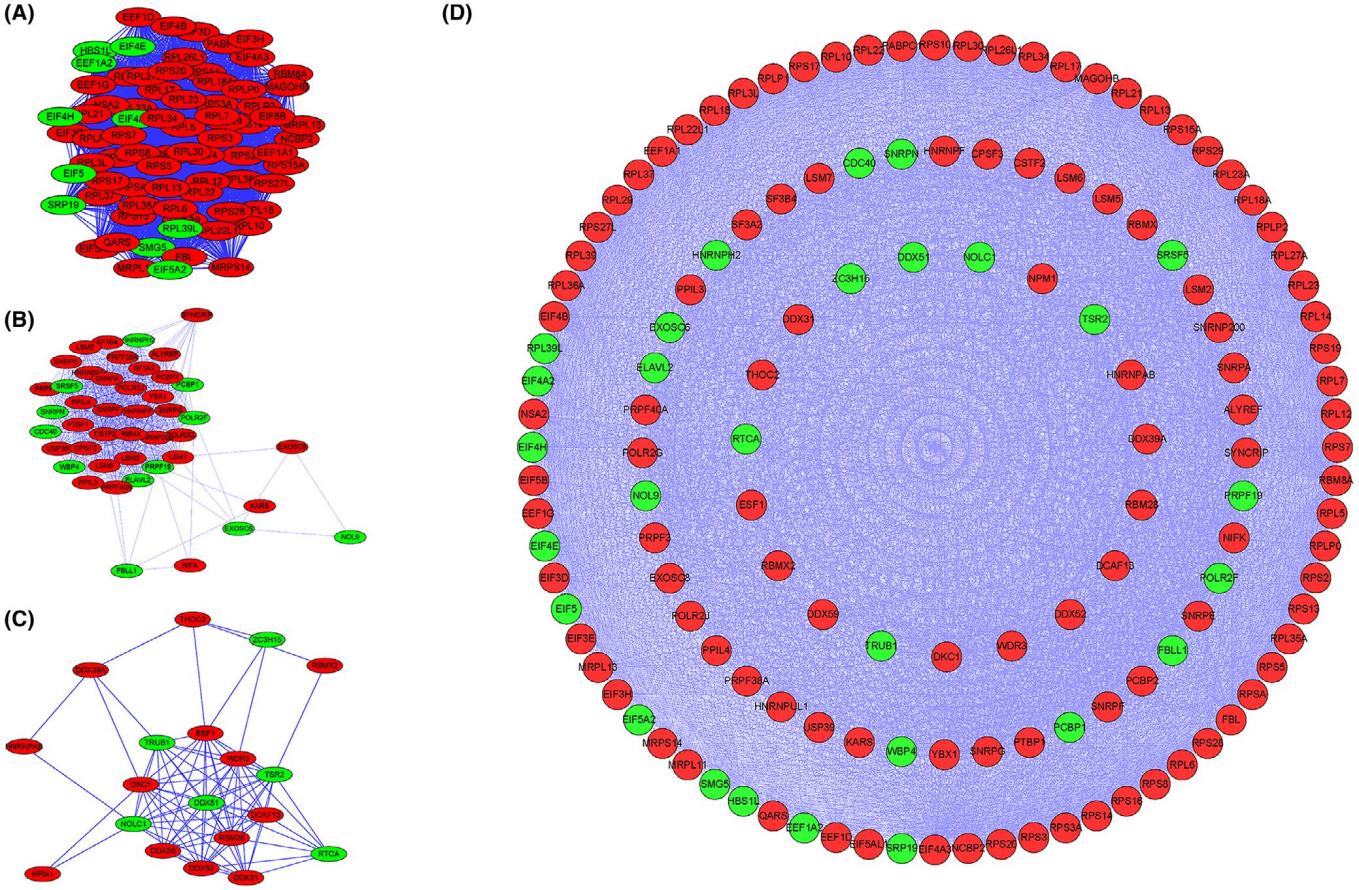
The RBPs differentially expressed between normal samples and glioma ones can be classified into three subnets according to their correlation coefficient. (A) Subnet1 contains the RBPs which have the strongest association with others, (B) followed by Subnet2, (C) the RBPs in Subnet3 have the weakest correlation. (D) The relationship of these three subnets is shown. The RBP in green are downregulated in glioma while the ones in red are upregulated

After analyzing the correlation between different RBPs and glioma, we intended to find out the intrinsic connection of RBPs selected, so as to screen the core RBPs in gliomagenesis and development. The "interaction relationship prediction" of the selected genes was performed using the String database to obtain the correlation coefficient of these genes basing on existing research. Then Cytoscape analysis was used to obtain the core interaction network, dividing the differentially expressed genes into three subnets, respectively, called subnet1/2/3. RBPs in Subnet1 had the strongest correlation with others and they may be the most promising RBP therapeutic targets, followed by Subnet2, and the RBPs in Subnet3 were correlated most weakly. In order to show the relationship between RBPs and glioma more intuitively, we showed RBPs with elevated expression in glioma in red and the ones with reduced expression in green (Figure 2).

GO and KEGG analysis was performed for each group of RBPs to find out their main function. The most critical ones, the RBPs in subnet1, were closely associated with ribosome, nuclear‐transcribed mRNA catabolic process, and translational initiation. The RBPs in subnet2 were strongly linked to RNA splicing, while the ones in subnet3 had a close relationship with ribosome biogenesis. (Tables 1 and 2) The regulation of cell activity by RBPs was mainly accomplished by regulating the structure and function of ribosome, as well as the post‐transcriptional process, such as RNA splicing and RNA degradation, thus affecting the synthesis of proteins and thus the biological function of cells, which may be of great significance in the tumorigenesis of glioma and provide new targets for glioma treatment.

**TABLE 1:** GO analysis for three subnets, respectively

Ontology	Description	*p*.adjust	*q* value
Subnet1			
BP	nuclear‐transcribed mRNA catabolic process, nonsense‐mediated decay	2.14E‐90	1.76E‐90
BP	translational initiation	2.75E‐88	2.26E‐88
BP	nuclear‐transcribed mRNA catabolic process	2.24E‐79	1.84E‐79
CC	cytosolic ribosome	8.57E‐89	4.14E‐89
CC	ribosomal subunit	2.60E‐83	1.26E‐83
CC	ribosome	5.34E‐77	2.58E‐77
MF	structural constituent of ribosome	5.44E‐79	3.74E‐79
MF	translation regulator activity	2.69E‐27	1.85E‐27
MF	translation regulator activity, nucleic acid binding	1.09E‐22	7.46E‐23
Subnet2			
BP	RNA splicing, via transesterification reactions with bulged adenosine as nucleophile	3.40E‐55	1.99E‐55
BP	mRNA splicing, via spliceosome	3.40E‐55	1.99E‐55
BP	RNA splicing, via transesterification reactions	3.40E‐55	1.99E‐55
CC	Spliceosomal complex	1.70E‐38	6.51E‐39
CC	U2‐type spliceosomal complex	5.32E‐28	2.04E‐28
CC	Catalytic step 2 spliceosome	5.10E‐24	1.96E‐24
MF	Single‐stranded RNA binding	0.000223	0.000159
MF	DNA‐directed 5'‐3′ RNA polymerase activity	0.00228	0.001623
MF	Single‐stranded DNA binding	0.00228	0.001623
Subnet3			
BP	Ribosome biogenesis	6.98E‐12	4.04E‐12
BP	Ribonucleoprotein complex biogenesis	3.26E‐10	1.89E‐10
BP	rRNA processing	6.91E‐10	4.00E‐10
CC	Box H/ACA RNP complex	0.001199	0.000705
CC	Transcription export complex	0.00121	0.000712
CC	Small nucleolar ribonucleoprotein complex	0.003325	0.001956
MF	RNA helicase activity	8.25E‐07	5.11E‐07
MF	Catalytic activity, acting on RNA	2.28E‐06	1.41E‐06
MF	Helicase activity	1.11E‐05	6.88E‐06

Abbreviations: BP, biological process; CC, cell cycle; MF, molecular function.

**TABLE 2:** KEGG analysis for three subnets, respectively

Description	*p*.adjust	*q* value
Subnet1		
Ribosome	9.21E‐70	7.27E‐70
RNA transport	2.95E‐10	2.33E‐10
mRNA surveillance pathway	0.000113	8.96E‐05
Subnet2		
Spliceosome	5.99E‐29	3.88E‐29
RNA degradation	2.85E‐06	1.84E‐06
Subnet3		
Ribosome biogenesis in eukaryotes	3.00E‐05	2.11E‐05

### 3.3 11 most clinically relevant RBPs are used to establish a mathematical model to judge the clinical prognosis of glioma patients

**FIGURE 3:**
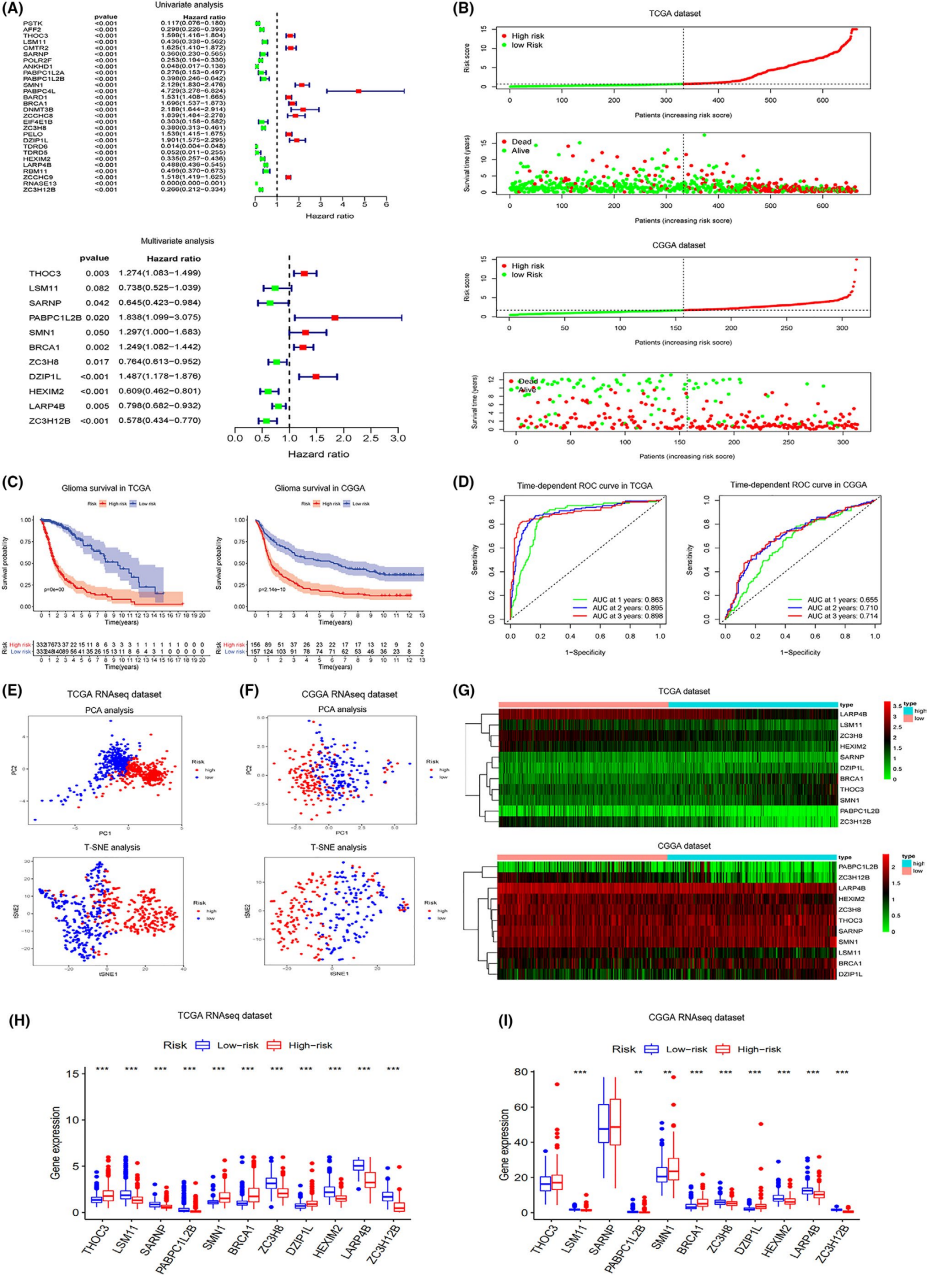
Model establishment and survival analysis of TCGA dataset and CGGA dataset. (A) 28 RBPs are selected after KM analysis (KM filter = 0.001), univariate Cox regression analysis (FDR filter = 0.001), and HR (Hazard Ratio) filter are set as 1.5 or 0.5. Eleven most clinically relevant RBPs are obtained to establish mathematical model by multi‐Cox analysis. (B) In TCGA and CGGA datasets, samples are divided into high‐risk group and low‐risk group bounded by the median risk score (0.712 in TCGA dataset and 1.692 in CGGA dataset). (C) Significant survival difference were observed between two groups in both TCGA and CGGA datasets, patients with higher risk score tend to suffer from poorer prognosis and survival. (D) The ROC curves for glioma patients, which predict survival according to risk score in TCGA and CGGA datasets. (E, F) PCA and t‐SNE of samples from TCGA and CGGA datasets, showing that there is a significant difference between high‐risk and low‐risk groups. (G) Heatmaps of the 11 RBPs for high‐risk and low‐risk groups in TCGA and CGGA datasets. (H, I) Boxplots of the 11 RBPs for high‐risk and low‐risk groups in TCGA and CGGA datasets. Kruskal–Wallis *H*‐test was used to compare difference between groups. ns: *p* > 0.05, *p < 0.05, **p < 0.01, ***p < 0.001

In order to further explore the relationship between RBP and glioma and provide ideas for the clinical application of RBP research, Kaplan–Meier Curve (KM) analysis (KM filter = 0.001) and univariate Cox regression analysis (FDR filter = 0.001) were used to select the RBPs that were clinically relevant from hundreds of them. Then, to select the RBPs which had the strongest clinical correlation, HR (Hazard Ratio) filter were set as 1.5 or 0.5 and 28 RBPs are obtained. To make our model more accurate, the multi‐Cox analysis was carried out, and 11 RBPs with the most clinical prognostic significance were extracted from the 28 RBPs mentioned to establish a mathematical model, including THOC3, LSM11, SARNP, PABPC1L2B, SMN1, BRCA1, ZC3H8, DZIP1L, HEXIM2, LARP4B, and ZC3H12B. The coefficient of them, respectively, are provided in Table 3. Among them, 6 RBPs’ hazard ratios were less than 0.5, while the other 5 have hazard ratios of more than 1.5 (Figure 3A).

**TABLE 3:** Coefficients of the 11 RBPs involved in the mathematical model

ID	coef	HR	*p* value
THOC3	0.242	1.274	0.0035
LSM11	−0.303	0.738	0.0821
SARNP	−0.438	0.645	0.0417
PABPC1L2B	0.609	1.838	0.0204
SMN1	0.260	1.297	0.0504
BRCA1	0.222	1.249	0.0024
ZC3H8	−0.269	0.764	0.0167
DZIP1L	0.397	1.487	0.0008
HEXIM2	−0.497	0.609	0.0004
LARP4B	−0.226	0.798	0.0045
ZC3H12B	−0.548	0.578	0.0002

To verify the model proposed, we downloaded RNA sequences of additional 325 glioma patients from CGGA and used our model to calculate the risk score for all patients in both TCGA dataset and CGGA dataset. In each dataset, we divided the patients into high‐risk group and low‐risk group, bounded by the median risk score (0.712 in the TCGA dataset and 1.692 in the CGGA dataset) (Figure 3B). It was obvious that the patients with higher risk score tended to have poorer prognosis and survival and there was a significant survival difference between the two groups in both TCGA and CGGA datasets (Figure 3C). The ROC curve suggested that our model had relatively high reliability in predicting the prognosis of glioma patients, with the AUC were 0.863 (TCGA, 1‐year OS), 0.898 (TCGA, 3‐year OS), 0.655 (CGGA, 1‐year OS), 0.714 (CGGA, 3‐year OS) (Figure 3D).

**FIGURE 4:**
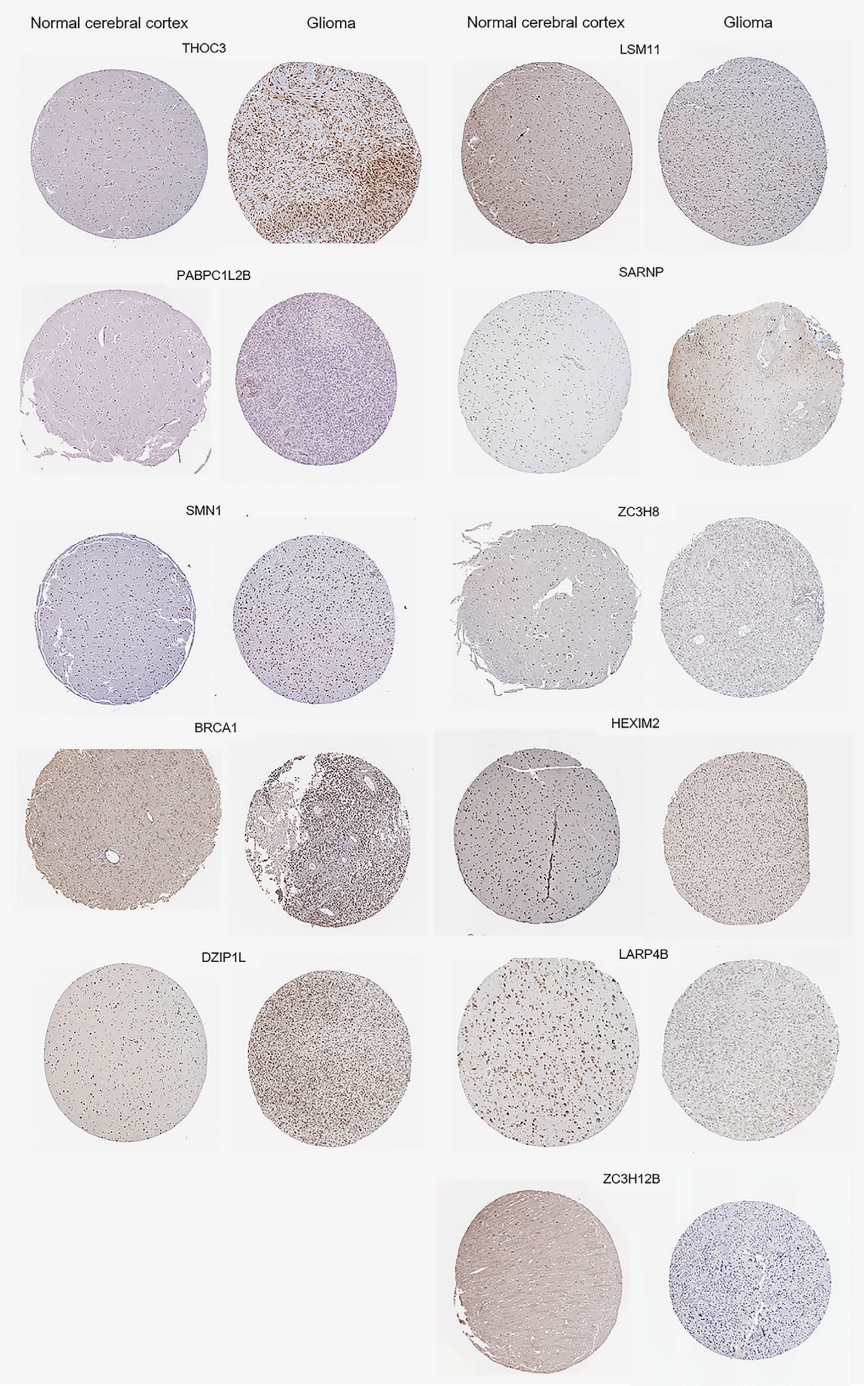
The slice images for differential expression of the 11 RBPs mentioned were obtained from the Human Protein Atlas. The staining results were basically consistent with our analysis, namely, RBPs presented on the left including THOC3, PABPC1L2B, BRCA1, DZIP1L, and SMN1 were up‐regulated in glioma, while the expression of the remaining six RBPs on the right was downregulated

**FIGURE 5:**
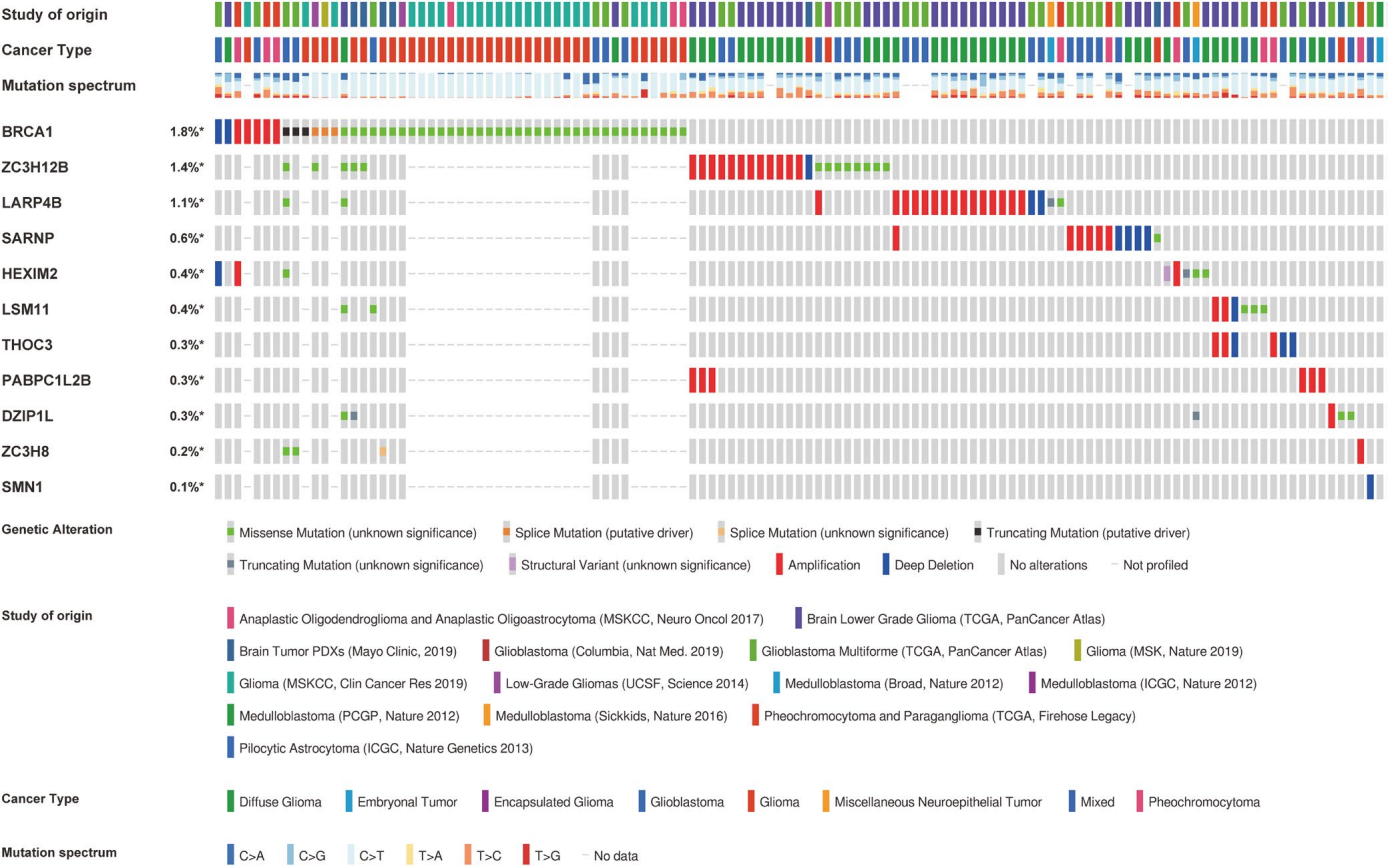
The oncoprint plot describing the mutation, copy number alteration, and structural variant landscape of the 11RBPs in glioma. The data was captured by exploiting the data from 14 different datasets correlated with glioma. The legend of different rectangles representing different genetic alterations, study of origins, cancer types, and mutation spectra were listed below

Principal Component Analysis (PCA) and t‐Distributed Stochastic Neighbor Embedding (t‐SNE) were performed to simplify and visualize the difference between the high‐risk group and low‐risk group, and significant differences were found, suggesting that our model is effective in predicting the prognosis of glioma patients (Figure 3E,F). In addition, it was evident that the expression of the selected 11 RBPs was discrepant between two groups no matter in the TCGA dataset or CCGA dataset (Figure 3G–I). We found the expressions of these 11 RBPs in normal cerebral cortical tissue and glioma from Human Protein Atlas, and found that their differential expression was basically consistent with our analysis (Figure 4). RBPs in the left column were upregulated in the glioma cerebral cortex, while the ones in the right columns were downregulated. Additionally, we also exploited the data from 14 different glioma datasets to construct the landscape of the mutation, copy number alteration, and the structural variant of these 11 RBPs. Data showed that the alteration of genomic alteration was more common in BRCA1, ZC3H12B, and LARP4B (Figure 5).

### 3.4 Immune function is critical for the risk grading of glioma patients

**FIGURE 6:**
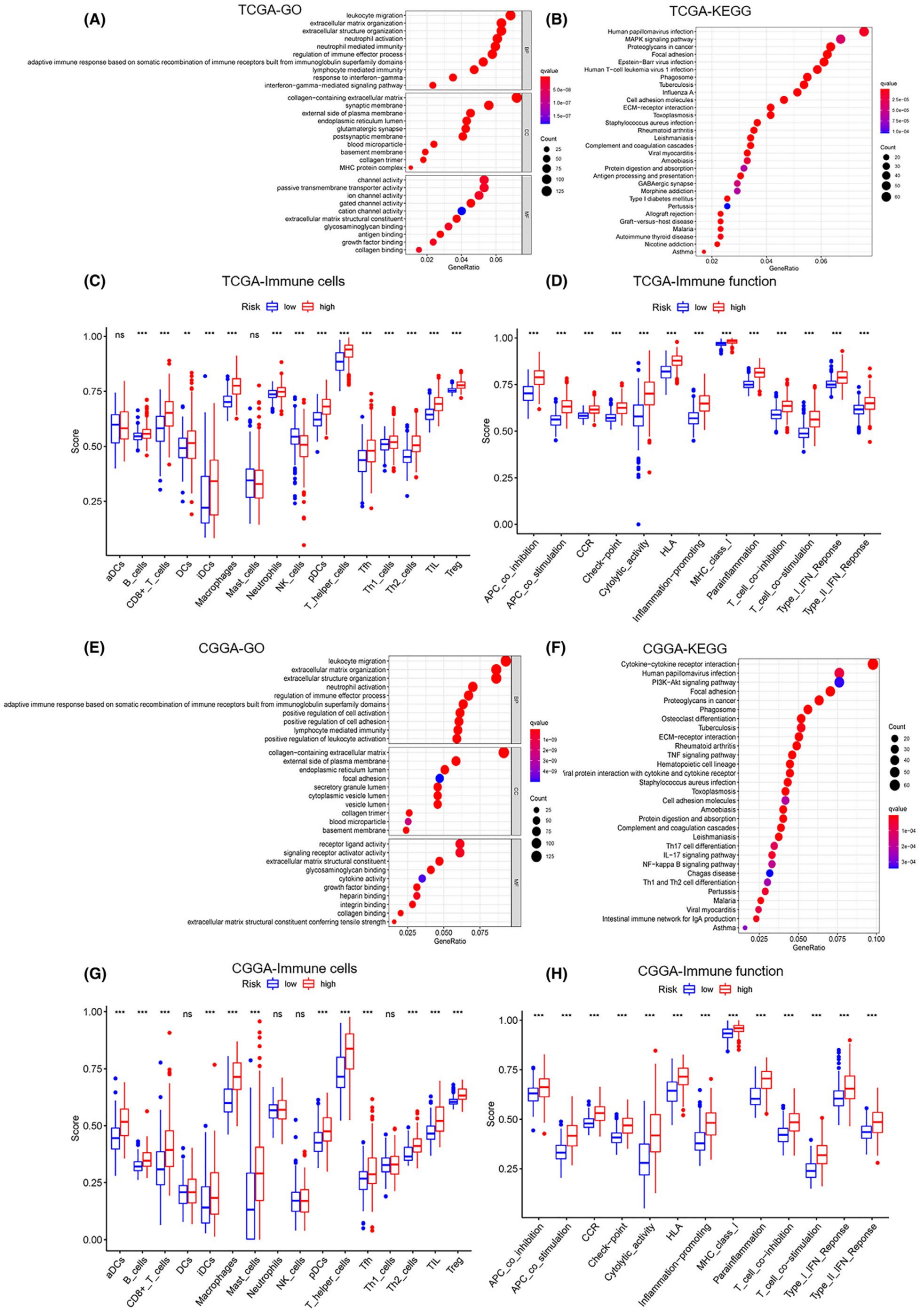
Pathway analysis showed a high association between RBPs and tumor microenvironment immune function (A) GO analysis of differentially expressed genes between high‐risk and low‐risk groups in the TCGA dataset. (B) KEGG analysis of differentially expressed genes between high‐risk and low‐risk groups in TCGA dataset. (C) Boxplot of comparison of immune cells between high‐risk and low‐risk groups in TCGA dataset. (D) Boxplot of comparison of the immune system between high‐risk and low‐risk groups in TCGA dataset. (E) GO analysis of differentially expressed genes between high‐risk and low‐risk groups in the CGGA dataset. (F) KEGG analysis of differentially expressed genes between high‐risk and low‐risk groups in CGGA dataset. (G) Boxplot of comparison of immune cells between high‐risk and low‐risk groups in CGGA dataset. (H) Boxplot of comparison of the immune system between high‐risk and low‐risk groups in CGGA dataset. Kruskal–Wallis *H*‐test was used to compare the the difference between groups. ns: *p* > 0.05, *p < 0.05, **p < 0.01, ***p < 0.001

**FIGURE 7:**
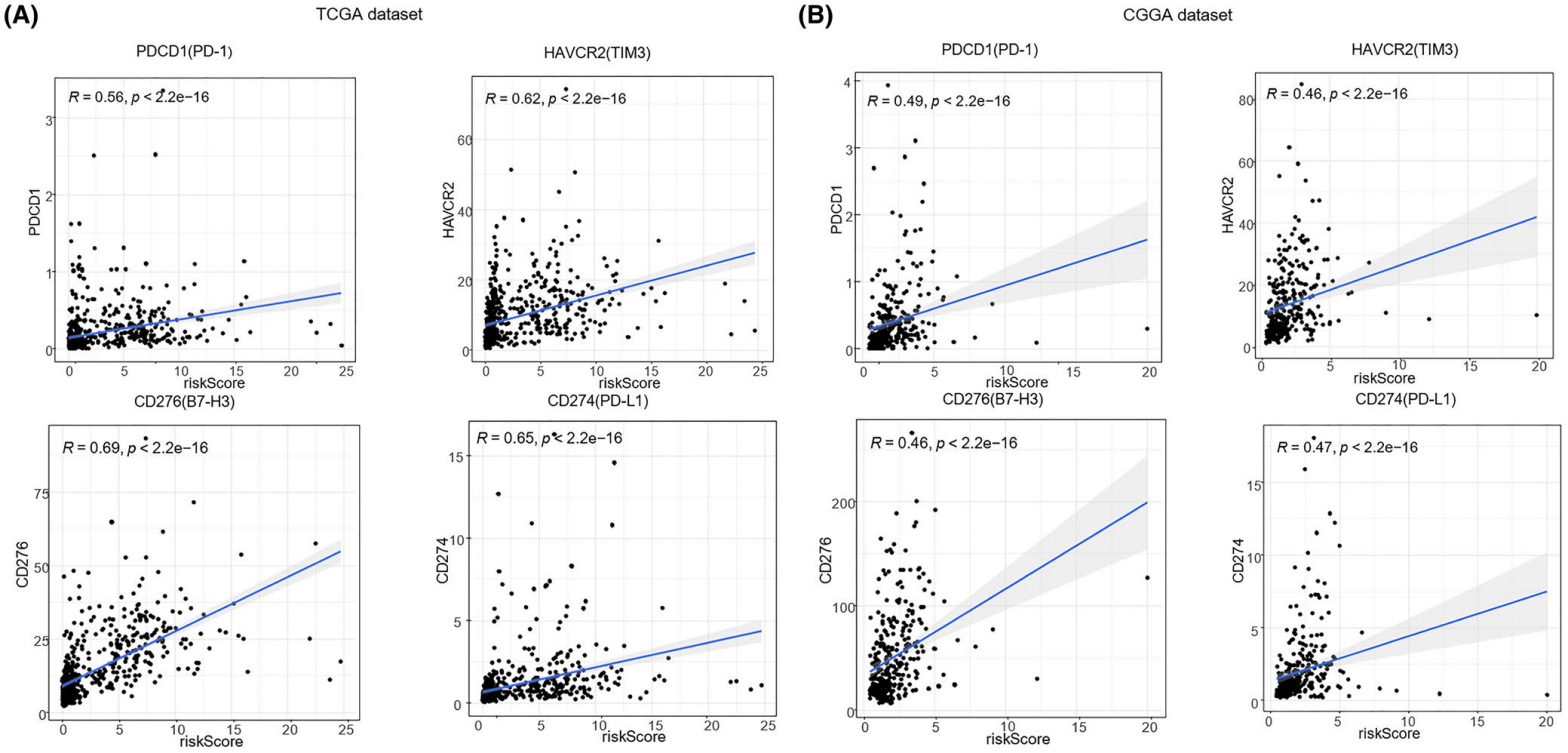
Immunosuppression was positively correlated with risk score in glioma. (A) Correlation between risk score and marker immunosuppressive genes’ expression in glioma patients of TCGA dataset, including PDCD1(PD‐1), HAVCR2 (TIM3), CD274(PD‐L1), CD276(B7‐H3). (B) Correlation between risk score and marker immunosuppressive genes’ expression in glioma patients of CGGA dataset, including PDCD1(PD‐1), HAVCR2 (TIM3), CD274(PD‐L1), CD276(B7‐H3). Spearman rank correlation test was utilized to estimate the strength of correlation. *p* < 0.05 was considered as statistically significant

Studies have found that the immune system played an important role in glioma development. It has been reported that GBM cells could establish an immune‐privileged microenvironment by releasing extracellular vesicles to transfer immune‐modulating molecules to immune cells, and macrophages could be recruited to GBM through the mediation by osteopontin.^34^, ^35^ We inferred that there may be similar mechanisms mediated by RBPs, which participated in the incidence of glioma by disturbing immune function. To confirm this, we performed GO and KEGG analysis of differentially expressed genes between high‐risk and low‐risk groups in TCGA and CGGA datasets and found that most of them were related to immunity. The regulatory targets included cytokine−cytokine receptor interaction, leukocyte migration, extracellular matrix organization, human papillomavirus infection, and so on (Figure 6A,B,E,F). This indicated that the prognosis of glioma patients was related to the function of their immune system. We calculated immune scores for both groups to explore changes in immune cells and immune function in glioma. In the high‐risk group, the scores of a variety of immune cells were obviously increased, including, T‐helper cells, macrophages, CD8+ T cells, iDCs (immature dendritic cells), pDCs (precursor DCs), Th2 cells, TIL (tumor‐infiltrating lymphocytes), and regulatory T cells (Treg). The immune function of the high‐risk group was also upregulated, particularly in cytolytic activity, human leukocyte antigen (HLA), inflammation‐promoting, antigen‐presenting cells (APC) co‐stimulation, and APC co‐inhibition (Figure 6C,D,G,H). We found that the immune systems of the high‐risk group patients were more active, but the survival of high‐risk patients worsened. We then calculated the correlation between risk score and marker immunosuppressive genes’ expression in glioma patients and found that there was a positive correlation between them, indicating that the more immune cells and the more immune function was activated, the greater the intensity of immunosuppression (Figure 7). As a result, regulating the immune function of glioma patients may improve their survival. Several studies about the immune system and glioma therapeutic approaches were ongoing, including the dendritic cells‐based immunization approach and therapeutic modulation of phagocytosis in GBM.^36^, ^37^

### 3.5 The values of our model on glioma prognosis prediction and novel drug identification may be useful for clinical treatment

**FIGURE 8:**
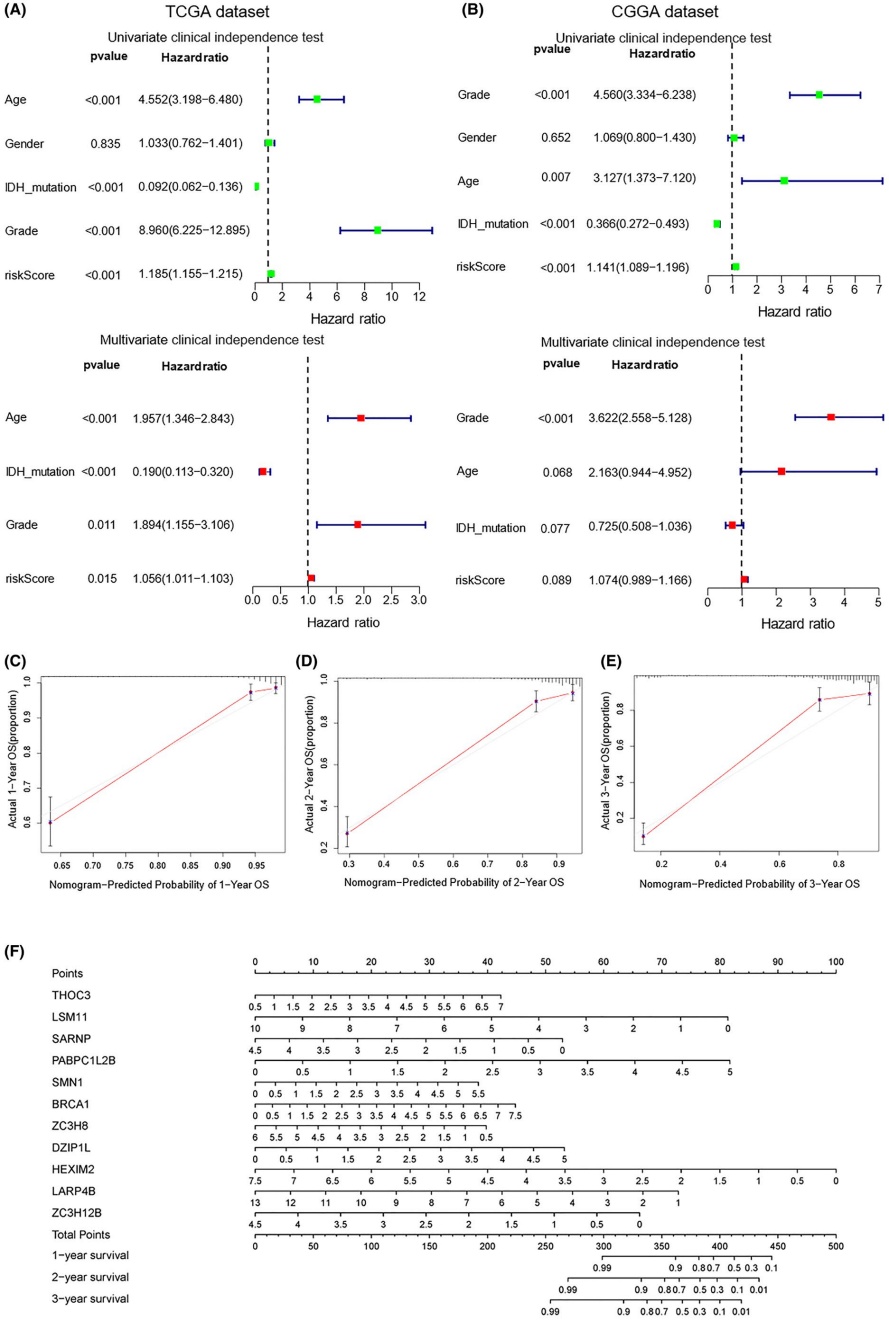
The validation of the clinical independence of RBP‐related prognostic model. (A, B) Clinical independence test for our model with data from both TCGA and CGGA datasets. (C–E) Nomograms (1–3 years) for verifying the formula, the curve calculated by our formula is in good agreement with the calibration line. (F) A clinical predictive nomogram based on the risk score calculating formula

To confirm the effectiveness of the mathematical model mentioned, we performed a clinical independence test for our model with data from both TCGA and CGGA datasets. The risk score calculated by the formula brought up by us was relevant with age, glioma grade, and IDH mutation but had little correlation with gender (Figure 8A,B). We established nomograms (1–3 years) to verify the formula, qualitative analysis showed that our model is effective in predicting the 1–3 years survival rate of glioma patients as the curve calculated by our formula was in good agreement with the calibration line (Figure 8C–E). We presented a clinical predictive nomogram based on the risk score calculating formula mentioned and hope it can provide a reference for the clinical diagnosis and grading of glioma patients (Figure 8F).

**FIGURE 9:**
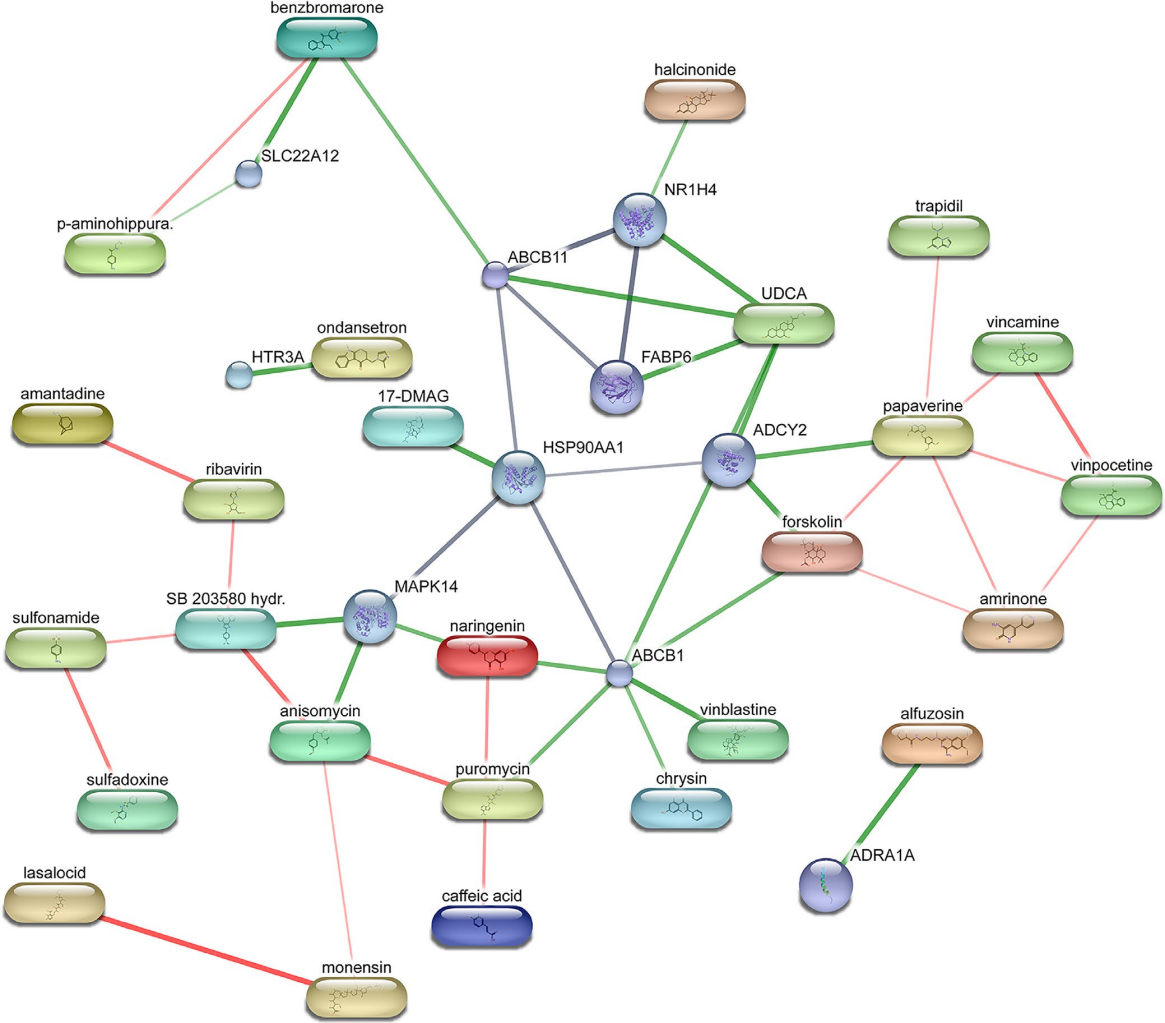
Potential targets and regulatory pathways of the drugs selected from the Cmap database. The red lines show drug‐drug interactions, and green lines represent interactions between the drug and the target protein, while the grey ones mean protein‐protein interaction. The thickness of the line indicated the correlation strength between the two substances

Moreover, according to the genes with differential expression in glioma, we searched for drugs that may be useful for glioma treatment by calculated in the Cmap database and they are demonstrated in Table 4. The drugs we screened included anisomycin, puromycin, 16‐phenyl tetranor prostaglandin E2, 5182598, sulfadoxine, and NU‐1025. Since these drugs were screened based on the same data analysis, we suspected that they may have similar mechanisms and targets. In order to figure out the potential targets and regulatory pathways of these drugs, we selected drugs with *p* value <0.05 to study the proteins targeted by them using the STITCH database (Figure 9). We identified a number of important downstream regulatory proteins, including MAPK14, HSP90AA1, ABCB11, FABP6, ABCB1, NR1H4, ADCY2, etc.

**TABLE 4:** Drugs can be potentially used in glioma treatment

Rank	Cmap name	Mean	Enrichment	*p* value	Specificity
1	anisomycin	−0.685	−0.942	0	0.0254
2	puromycin	−0.643	−0.914	0.00010	0.0299
3	16‐phenyltetranorprostaglandin E2	−0.596	−0.905	0.00014	0
4	5182598	−0.744	−0.978	0.00107	0.0126
5	sulfadoxine	−0.667	−0.905	0.00162	0
6	NU‐1025	−0.529	−0.908	0.01708	0.0161

The values of mean and enrichment were the indicators to measure the effectiveness of drugs, and the absolute value was positively correlated with the therapeutic effect of drugs, while the negative value represented its inhibitory effect on tumorigenesis.

### 3.6 IC50 analyses based on RBP‐related signature showed different drug sensitivities between high‐risk and low‐risk groups

**FIGURE 10:**
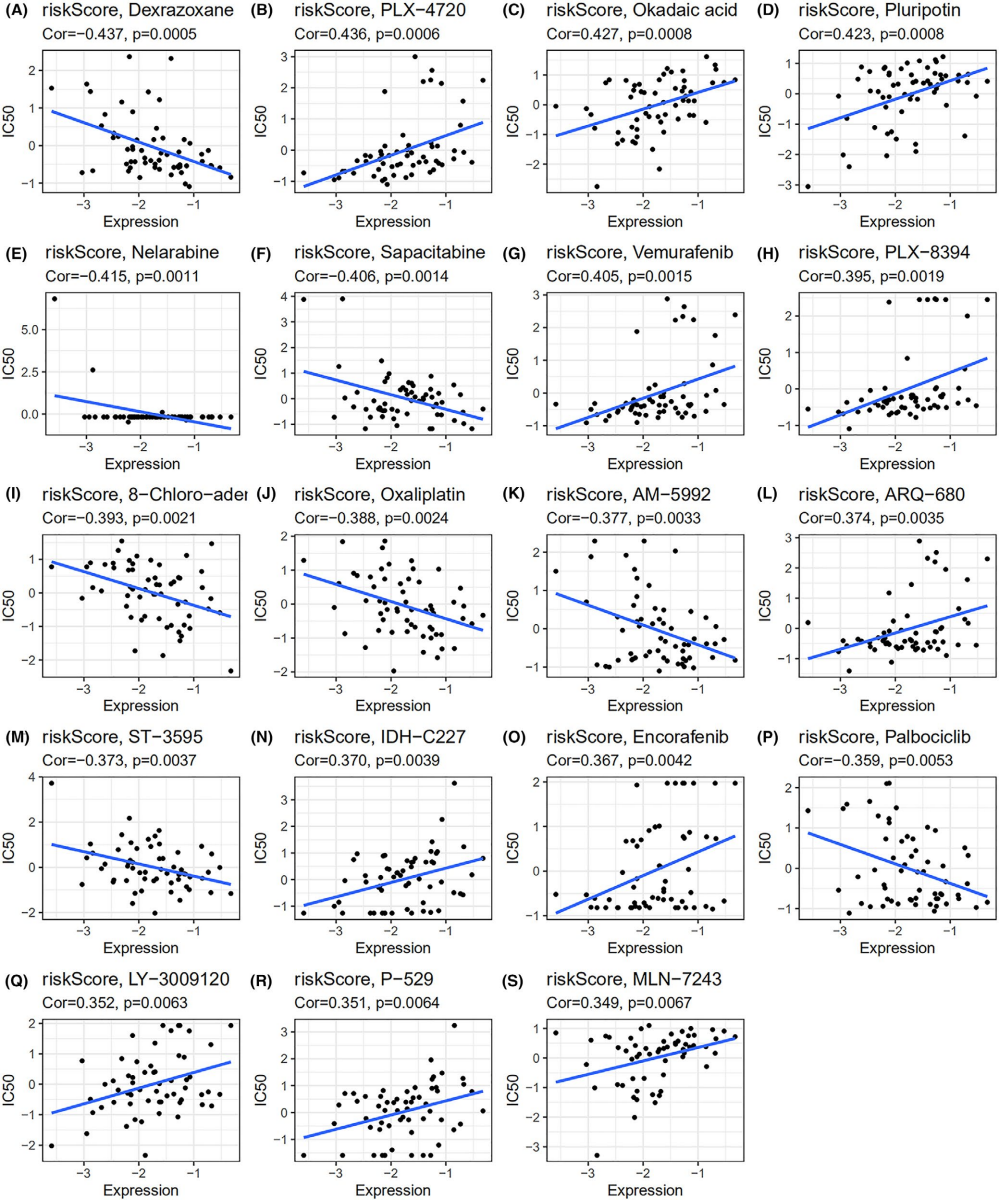
Correlations between cell risk score and IC50 for different drugs. The correlation analysis between RBP‐related signature‐based risk score and IC50 of (A) Dexrazoxane, (B) PLX‐4720, (C) Okadaic acid, (D) Pluripotin, (E) Nelarabine, (F) Sapacitabine, (G) Vemurafenib, (H) PLX‐8394, (I) 8‐Chloro‐adenosine, (J) Oxaliplatin, (K) AM‐5992, (L) ARQ‐680, (M) ST‐3595, (N) IDH‐C227, (O) Encorafenib, (P) Palbociclib, (Q) LY‐3009120, (R) P‐529, (S) MLN‐7243. Spearman rank correlation test was implemented to estimate the correlation between risk score and IC50 of each drug. *p* <  0.05 was considered as statistically significant

**FIGURE 11:**
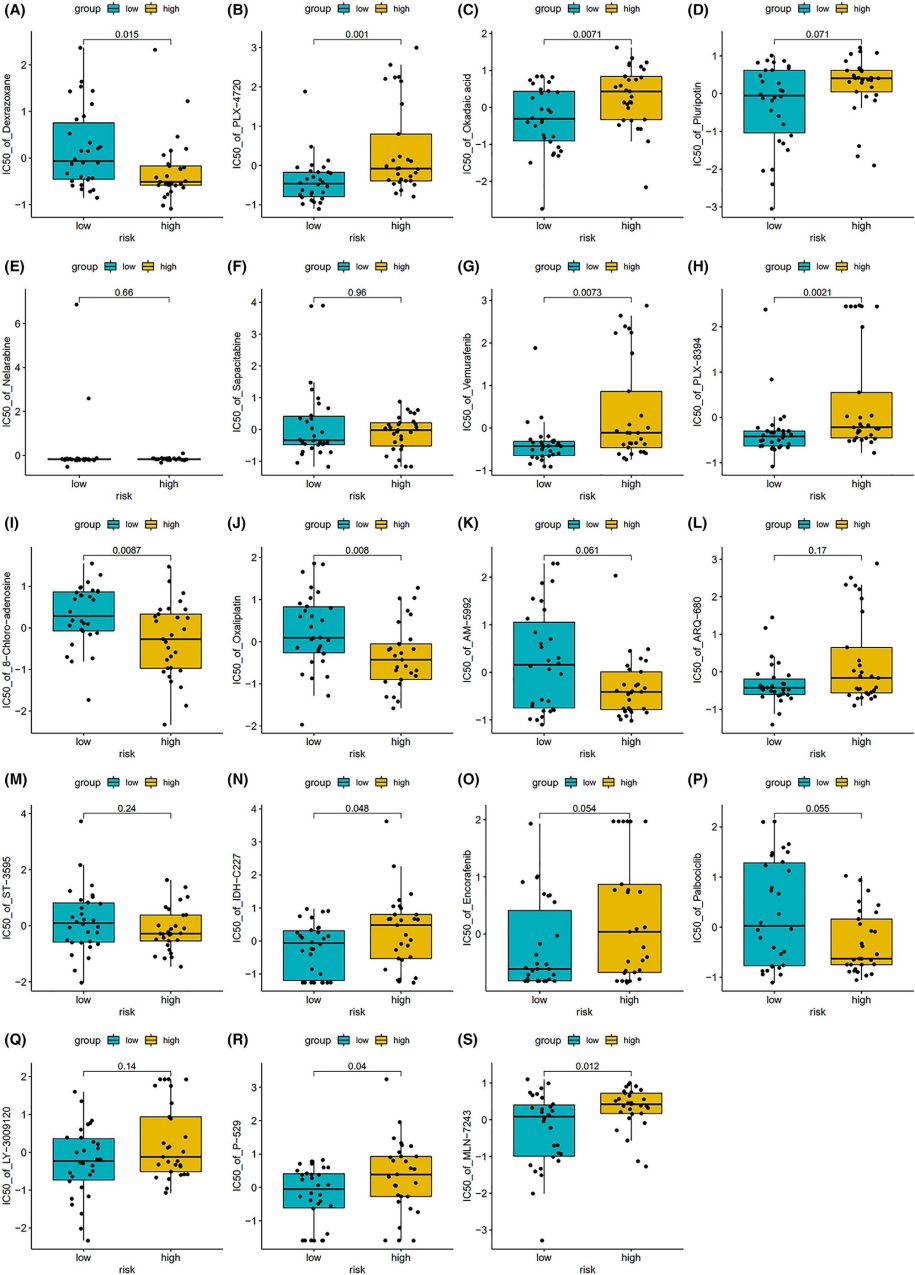
Comparing the efficiency of the selected drugs in high‐risk group and low‐risk group. (A) Dexrazoxane, (B) PLX‐4720, (C) Okadaic acid, (D) Pluripotin, (E) Nelarabine, (F) Sapacitabine, (G) Vemurafenib, (H) PLX‐8394, (I) 8‐Chloro‐adenosine, (J) Oxaliplatin, (K) AM‐5992, (L) ARQ‐680, (M) ST‐3595, (N) IDH‐C227, (O) Encorafenib, (P) Palbociclib, (Q) LY‐3009120, (R) P‐529, (S) MLN‐7243. Kruskal–Wallis *H*‐test was used to compare the difference between groups. *p* < 0.05 was considered as statistically significant

In order to further prove that the RBP‐based risk score established by us has good reliability, we studied the drug sensitivity of the compounds selected according to this model. We used our risk score calculating model to screen out these compounds by an analysis on the transcriptome for 60 cancer cell lines collected from the CellMiner database, which included five glioma cell lines, and data of IC50 (50% inhibitory concentration) of over 20,000 compounds. To better link our RBP‐related signature to the clinical practice, we only involved 707 drugs that have been approved by FDA or are now under clinical trials. First, we calculated the risk score of the mentioned cell lines medicated by different drugs. Then for each kind of drug, we analyzed the relationship between risk score and IC50, and thus filtered out several drugs whose IC50s were strongly correlated with the risk score (Figure 10). Statistically significant correlations were identified with |cor| > 0.3 and *p* value <0.01. We also performed a comparative study on these drugs between high‐risk group and low‐risk group, and the IC50 of drugs showed a significant difference between the two groups, such as okadaic acid, PLX‐4720, PLX‐8394, vemurafenib, oxaliplatin and 8‐Chloro‐adenosine, which means that our risk score calculation model was effective in predicting the sensitivity of cancer cells to these drugs (Figure 11). It also demonstrated that we can select drugs based on a patient's risk score, which may lead to more precise drug use.

## 4 DISCUSSION

RBPs play an important role in post‐transcriptional regulation and the activity of RBP‐RNA networks has been shown to be closely related to tumor development, so the differential expression of RBP can be used as a basis for tumor typing and tumor malignancy, and can be used to predict the prognosis of patients.^9^

In this study, we screened out genes with significant differential expression between glioma samples and normal samples based on TCGA‐GBM, TCGA‐LGG, and CGGA datasets, and through univariate and multivariate Cox, we screened out 11 RBPs that can provide a reference for the prognosis prediction of glioma, then established a model and a rigorous verification was carried out. These 11 RBPs were closely linked to metabolism and glioma development. THOC3 was involved in the THO subcomplex, which was necessary for coupled mRNA transcriptional extension and nuclear export, its expression was significantly elevated in glioma cells.^38^ LSM11 participated in histone RNA 3’ processing and SARNP, playing a role in mRNA splicing and export, was not only significant in glioma but also acted as an important molecule in triple‐negative breast cancer.^39^, ^40^ Both LSM11 and SARNP played roles in preventing disorders of cell protein synthesis or cell division, and their decreased expression in gliomas may be associated with glioma development. BRCA1 and LARP4B were traditionally recognized as a tumor suppressor but BRCA1 unexpectedly plays as a promotor in GBM.^18^, ^41^ Our work illustrated this at the molecular level as BRCA1’s expression was significantly increased in glioma cells, while the expression of LARP4B was decreased. As members of ZC3H‐family, ZC3H8, and ZC3H12B could negatively regulate NFκB and affect cell proliferation, survival, and differentiation.^42^ HEXIM1 was a promiscuous double‐stranded RBP and participates in several biological processes including controlling P‐TEFb, which regulated eukaryotic gene expression.^43^ In this study, we synthesized the 11 RBPs which played important roles in the regulation of cell proliferation and metabolism in different aspects, and established a relevantly highly reliable prognosis prediction model for glioma patients, providing a reference for the grading of risk score and the selection of clinical treatments.

We also found that those RBPs with differential expression were closely related to immune cells and immune function. Interestingly, in high‐risk glioma patients, the immune gene sets associated with immune promoting (TIL, CD8+ T cells, etc.) and immune‐suppressing (Treg, APC co‐inhibition, etc.) tumor immunity were both upregulated, but the prognosis was worse in high‐risk glioma patients as immunosuppression was positively correlated with risk score.

The increase of immune cells and function in glioma was associated with chemokine in tumor tissue. For instance, CD8+ cells and chemokine CCL5 were both observed increased in GBM, CCL5‐CCR5 axis may be an important mechanism for attracting effector T cells such as CD8+ T cells from tumor tissue to tumor microenvironment. Being activated in glioma, CCR5+CD38+HLA‐DR+CD8+ T cells express a higher level of PD‐1, suggesting that the PD‐1/PD‐L1 loop may be a potential target for glioma treatment.^44^ However, it has been reported that glioma cells could lead to suppression of the immune system. By recruiting innate immune cells, inducing their phenotype modification and suppressing adaptive immune responses, glioma tumor cells could downregulate antitumor response. Researchers have also found reduced Neoantigen expression, which may contribute to the inhibited immune function.^45^ In addition, tumors also induced normal brain cells to create a microenvironment suitable for tumor proliferation and invasion of brain.^46^ What is more, it has been reported that myeloid‐derived suppressor cells (MDSC), which was elevated in peripheral blood in glioma patients, were more tend to differentiate into DCs in LGG and remain MDSC in GBM, this may also be related to the induction of immune cells by high‐grade gliomas.^47^

Upregulation of tumor suppressor and immune‐related genes did not necessarily improve the prognosis of patients, which may be related to the concurrent intensification of immunosuppression. Through computational analysis, we found that the increase of risk score was usually accompanied by the enhancement of immunosuppression as a variety of marker immunosuppressive genes’ expression present positively correlative with risk score. In addition, glioma cells had the ability to induce immune cells to evade immune examination and induce normal nerve cells to participate in the establishment of tumor microenvironment, so the final manifestation in high‐risk glioma patients was immunosuppression and deterioration. Although the gene expressions related to TILs are elevated, exhaustion and clonal restriction were found in the TILs of patients with GBM, suggesting that inhibition was stronger than promotion to TILs.^48^ It has been reported that microglia cells and macrophages in the glioma microenvironment could secrete chemokine CCL2 to recruit CCR4+ Treg and CCR2+ ly‐6C+ Monocytic MDSCs and suppress tumor immunity.^49^ It is worth mentioning that increased gain‐of‐function mutation of TP53 in glioma promoted antitumor inflammation, but deteriorated prognostic outcomes in patients with GBM either because inflammation can accelerate the process of GBM or strengthen treatment resistance.^50^ This may be the reason why genes regulating immunity were generally up‐regulated but the prognosis of patients was poorer. Research is underway to overcome immunosuppression in gliomas. CXCL16/CXCR6 signaling acted directly on promoting tumor cell growth, migration and invasion by promoting glioma‐associated microglia/macrophages modulation toward a pro‐tumor phenotype, being a critical target for glioma treatment.^51^ Immunotherapy may provide a breakthrough for the treatment of glioma. Researchers have found that glioma cause changes of immune cells in the brain and therapeutic approaches that can target intracellular immune pathways have been brought up.^52^, ^53^

On the basis of the analysis of differentially expressed genes, we also selected several drugs that might be effective for the treatment of glioma and yielded their main target proteins. Anisomycin and puromycin, as protein synthesis inhibitors, could Inhibit tumor proliferation.^54^ Anisomycin induced glioma cell death via downregulation of the PP2A catalytic subunit.^55^ It is also a potential drug for immunotherapy in hepatocellular carcinoma by playing an important role in both direct killing and natural killer (NK) cell‐mediated immunotherapy.^37^ Puromycin‐based inhibitors of aminopeptidases have shown great potential in the treatment of hematologic malignancies. Sulfadoxine was traditionally used for the treatment of malaria, but some studies have found that it had a certain potential in the suppression of cancer. A double‐blind, placebo‐controlled study found that prophylactic use of trimethoprim—sulfadiazine reduced the incidence of osteosarcoma or lymphoma in dogs during the first 14 days after doxorubicin treatment.^56^ Benzarone has been confirmed in zebrafish experiments that it could inhibit tumor angiogenesis by inhibiting the activity of pro‐angiogenic tyrosine phosphatase Eyes Absents (EYA). However, single‐agent therapy has provided disappointing outcomes so far, the combination of a variety of therapies, especially biomarker‐targeted clinical trials will be the focus of future therapeutic development.^57^ NU‐1025, as a poly (ADP ribose) polymerase inhibitor, was basically inactive when used as a single drug, but it could significantly improve the effect of temozolomide (TMZ) on the treatment of malignant tumors in the brain of mice when used as a combination with TMZ.^58^

Correlation analysis of glioma risk score and drug sensitivity identified a number of drugs with different sensitivities in the high‐risk and low‐risk groups, the ones with significant difference (*p* value <0.01) included okadaic acid, PLX‐4720, PLX‐8394, vemurafenib, oxaliplatin and 8‐Chloro‐adenosine. This suggested that the selection of different drugs for patients with different risk scores may improve the outcome. It was reported that okadaic acid could induce apoptosis of malignant glioma cells through inhibiting dephosphorylation, which activated c‐Jun‐N‐terminal kinase (JNK) and extracellular signal‐regulated kinase (ERK) pathways to promote apoptosis.^59^ And our analysis pointed out that okadaic acid has a better therapeutic effect in low‐risk glioma patients. Vemurafenib and its analog PLX‐4720 were promising molecules for inhibiting tumor growth in gliomas with BRAF^V600E^ mutation, which was more common in children,^60^ and both of them showed a higher sensitivity in the low‐risk group. However, using BRAF^V600E^ inhibitors alone could easily induce glioma cells’ resistance to them, the escape mechanisms of glioma cells included the up‐regulation of the Wnt pathway and increased activity of receptor tyrosine kinases, including EGFR.^60^ And a combination of BRAF^V600E^ inhibitors and pharmacologic inhibition of EGFR could greatly improve the efficacy by decreasing glioma cell proliferation and promoting apoptosis.^61^ PLX‐8394 is a kind of drug with limited study, our analysis based on the database predicted that it may have better effectiveness in the low‐risk group. Oxaliplatin, which has a higher sensitivity to glioma with high‐risk score, has been proved to be a promising anti‐glioma drug as it inhibited glioma growth in a relatively low concentration.^62^ It increased glioma cell apoptosis through reactive oxygen species (ROS)‐dependent mitochondrial pathway by inhibiting the activity of signal transducer and activator of transcription 3 (STAT3) and downregulating the level of O‐6‐methylguanine‐DNA methyltransferase (MGMT).^62^ Further research found out that it had a synergistic activity with silver nanotriangles (AgNTs).^63^ 8‐Chloro‐adenosine has been shown to inhibit glioma cell proliferation by disrupting nucleic acid synthesis and block cell cycle at the G2 M phase,^64^ but the research of 8‐Chloro‐adenosin in glioma is still limited. Our research suggested that it may be more effective for high‐risk glioma treatment.

Immunotherapy is considered one of the most promising treatments for glioma and a lot of work is ongoing. Among various immunotherapies, vaccination was considered to be one of the most promising ways to improve the prognosis of patients with glioblastoma, but as single‐modality immunotherapy the challenge remained.^65^ Measures have been taken to improve the probability of successful immunotherapy, in order to improve the efficacy of antitumor immunity within a meaningful time window.^66^ Immune checkpoint was another important target for glioma treatment and some immunotherapy drugs had entered clinical trials, inhibition of PD‐L1, CTLA4, Indoleamine 2,3‐dioxygenase (IDO) was included.^67^ Many immunotherapy drugs have been listed as potential strategies for treatments of glioma, and we believe that immunotherapy will provide more possibilities for glioma treatment in the future.

However, due to the inevitable limitations of data sources and research methods, there are still some deficiencies in this study. We established the mathematic model by multi‐Cox analysis, which was more likely to retain some of the less weighted factors, but it also made our modeling results more comprehensive. Although the heterogeneity of the patients’ data rooting in patients’ different backgrounds would cause a difference between results from are TCGA dataset and CGGA dataset to some degree, but the similarities among them were statistically significant and were verified by various methods. In risk score‐IC50 correlation analysis, due to the limited data of glioma cell lines, we selected a part of non‐glioma cell lines for analysis, which may affect the accuracy of the results. But delightfully, the comparison between the high‐risk group and the low‐risk group proved that the results of the correlation analysis had good reliability. What is more, due to limited conditions, we have not verified our results at the cellular and animal levels, and we hope that further studies can be conducted in the future.

## 5 CONCLUSIONS

In general, we screened 11 RBPs with strong clinical prognostic relevance from hundreds of differentially expressed RBPs in glioma, and established a prognosis predicting model for glioma patients with them. We also found that RBPs differentially expressed in gliomas were closely related to immune cells and immune function, indicating that immunotherapy will be an important direction in glioma therapy. In addition, through the analysis of differentially expressed RBPs, we also selected several drugs and some possible drug regulatory targets that may be useful in the treatment of glioma based on these aberrantly expressed RBPs. In addition, we identified a number of drugs with different sensitivities between high‐risk and low‐risk glioma patients through risk scoring combined with drug sensitivity analysis. We hope that our work could provide a reference for the clinical prognosis and treatment of glioma.

## ETHICAL APPROVAL STATEMENT

Not applicable.

## CONFLICT OF INTEREST

The authors declare no conflict of interest.

## CLINICAL TRIAL REGISTRATION NUMBER

Not applicable.

## Data Availability

The datasets generated and analyzed during the current study are available in The Cancer Genome Atlas (TCGA) datasets, Chinese Glioma Genome Atlas (CGGA) database, Human Protein Atlas (HPA), and CellMiner database repository. The accessible websites of TCGA, CGGA, HPA and CellMiner are https://portal.gdc.cancer.gov/, http://www.cgga.org.cn/, https://www.proteinatlas.org/, and https://discover.nci.nih.gov/cellminer/home.do, respectively.
